# Intrathoracic rupture of amebic liver abscess: a case report and literature review

**DOI:** 10.1186/s41182-025-00809-2

**Published:** 2025-10-09

**Authors:** Kota Hasegawa, Akira Kawashima, Ryo Kuwata, Rieko Shimogawara, Mitsuko Sasaki, Yasuaki Yanagawa, Takato Nakamoto, Takahiro Aoki, Kenji Yagita, Koji Watanabe, Katsuji Teruya, Hiroyuki Gatanaga

**Affiliations:** 1 AIDS Clinical Center, National Center for Global Health and Medicine, Japan Institute for Health Security, 1-21-1 Toyama, Shinjuku-Ku, Tokyo Japan; 2 Department of Parasitology, National Institute of Infectious Diseases, Japan Institute for Health Security, 1-23-1 Toyama, Shinjuku-Ku, Tokyo Japan; 3 Joint Research Center for Human Retrovirus Infection, Kumamoto University, 2-2-1 Honjo, Kumamoto, Japan; 4 Department of Parasitology, Division of Host Defense Mechanism, Tokai University School of Medicine, 143 Shimokasuya, Isehara, Japan

**Keywords:** *Entamoeba histolytica*, Amebic liver abscess, Pleuropulmonary infection, Portal vein thrombosis, Case report

## Abstract

**Background:**

Amebic liver abscess (ALA) is a serious complication of *Entamoeba histolytica* infection. In rare cases, ALA may rupture into the thoracic cavity, leading to a high risk of death. Differentiating intrathoracic ALA rupture from reactive pleural effusion is essential for predicting the clinical course and appropriate management.

**Case presentation:**

A 46-year-old bisexual man with well-controlled human immunodeficiency virus infection presented with pain in the right shoulder and upper abdomen. Imaging revealed a solitary liver abscess with diaphragmatic rupture, right pleural effusion, and portal vein thrombosis. Results of stool microscopy, antigen testing, and cytology of pleural and liver aspirates were inconclusive. However, *E. histolytica* was identified in the stool, liver abscess aspirate, and pleural fluid using polymerase chain reaction tests. Despite the initial therapy with metronidazole, the thoracic fluid volume increased considerably, necessitating thoracic and hepatic drainage. After stabilization, anticoagulation therapy with edoxaban for portal vein thrombosis and luminal therapy with paromomycin were initiated. The patient showed progressive clinical improvement, and follow-up imaging confirmed shrinkage of the liver abscess and resolution of the thrombus and diaphragmatic rupture. No recurrence was observed during the 6-month follow-up period.

**Conclusions:**

We reported the case of a patient with a rapidly progressive ALA with intrathoracic rupture. In cases of ALA with thoracic rupture, performing drainage is important, considering that pleural effusion may progress rapidly. This case highlights the need for comprehensive management involving timely antimicrobial and anticoagulation therapy in cases of vascular thrombosis.

## Background

Amebiasis is the second most deadly parasitic disease after malaria [1], causing approximately 100,000 deaths annually [2]. Each year, 500 million people are infected, with approximately 10% of them harboring the pathogenic *Entamoeba histolytica* [3]. This disease is endemic in developing countries, where it is transmitted through contaminated water and food. However, in recent years, it has also emerged in developed countries owing to oroanal sexual contact, especially among men who have sex with men (MSM) [4]. The seroprevalence of *E. histolytica* in the high-risk population in Japan is reported to be 2.64% [5]. Additionally, cases of amebiasis have been reported among MSM in developed countries in Europe and East Asia [6–9]. Therefore, the possibility of amebiasis must be considered in this high-risk population.

Amebic liver abscess (ALA) is the most common form of extraintestinal amebiasis and is caused by the migration of *E. histolytica* trophozoites through the portal vein to the liver [1]. Typical symptoms include fever, cough, and dull pain in the epigastric or right upper quadrant, which usually progresses for 2–4 weeks. In addition, in patients with liver abscess rupture through the diaphragm into the thoracic cavity, pain may be referred to the shoulder owing to phrenic nerve irritation. The reported incidence of intrathoracic rupture of ALA ranges from 7 to 21% [10, 11]. Here, we report a case of ALA in which intrathoracic rupture was confirmed based on diaphragmatic disruption and pleural extension on imaging studies. This case emphasizes the importance of imaging and drainage in the diagnosis and treatment of intrathoracic rupture of ALA, respectively.

## Case presentation

A 40-year-old bisexual Japanese man presented to our emergency department with pain in the right shoulder and right upper quadrant of the abdomen. Two weeks before presentation, he experienced severe right shoulder pain and consulted a local orthopedist; however, no definitive diagnosis was made, and the patient was prescribed non-steroidal anti-inflammatory drugs for symptom relief. However, the pain worsened, and the patient was brought to our hospital by ambulance.

The patient was diagnosed with human immunodeficiency virus (HIV) infection 4 years earlier and was receiving antiretroviral therapy (bictegravir, emtricitabine, and tenofovir alafenamide) with sustained virological suppression. At the time of HIV infection diagnosis, his CD4 count was 385/μL, and no signs of immunodeficiency were noted. Additional medical history included chronic hepatitis B, condylomata acuminata, gonorrhea urethritis, and anal chlamydial infection. The patient reported no history of overseas travel.

On admission, the patient was fully conscious and alert. His vital signs were: temperature, 38.0 °C; blood pressure, 120/85 mmHg; heart rate, 111 beats per minute; respiratory rate, 18 breaths/min; and oxygen saturation, 97% on ambient air. Physical examination revealed tenderness and rigidity of the right upper abdomen, which prevented the assessment of hepatomegaly. Auscultation revealed decreased breath sounds in the right lower lung field and percussion demonstrated dullness in the same area, suggestive of right-sided pleural effusion.

Laboratory tests revealed leukocytosis (16,020/μL) with neutrophil predominance and a CD4-positive lymphocyte count of 476/μL. Additional findings included International Normalized Ratio of Prothrombin Time, 1.09; D-dimer, 2.6 μg/mL; aspartate aminotransferase, 46 U/L; alanine aminotransferase, 64 U/L; lactate dehydrogenase, 288 U/L; alkaline phosphatase, 148 U/L; γ-glutamyl transpeptidase, 99 U/L; C-reactive protein, 16.1 mg/dL. Serological testing for *E. histolytica* using enzyme-linked immunosorbent assay (Immuno-Biological Laboratories Co., Ltd., Japan) was positive at 25 units.

**Fig. 1:**
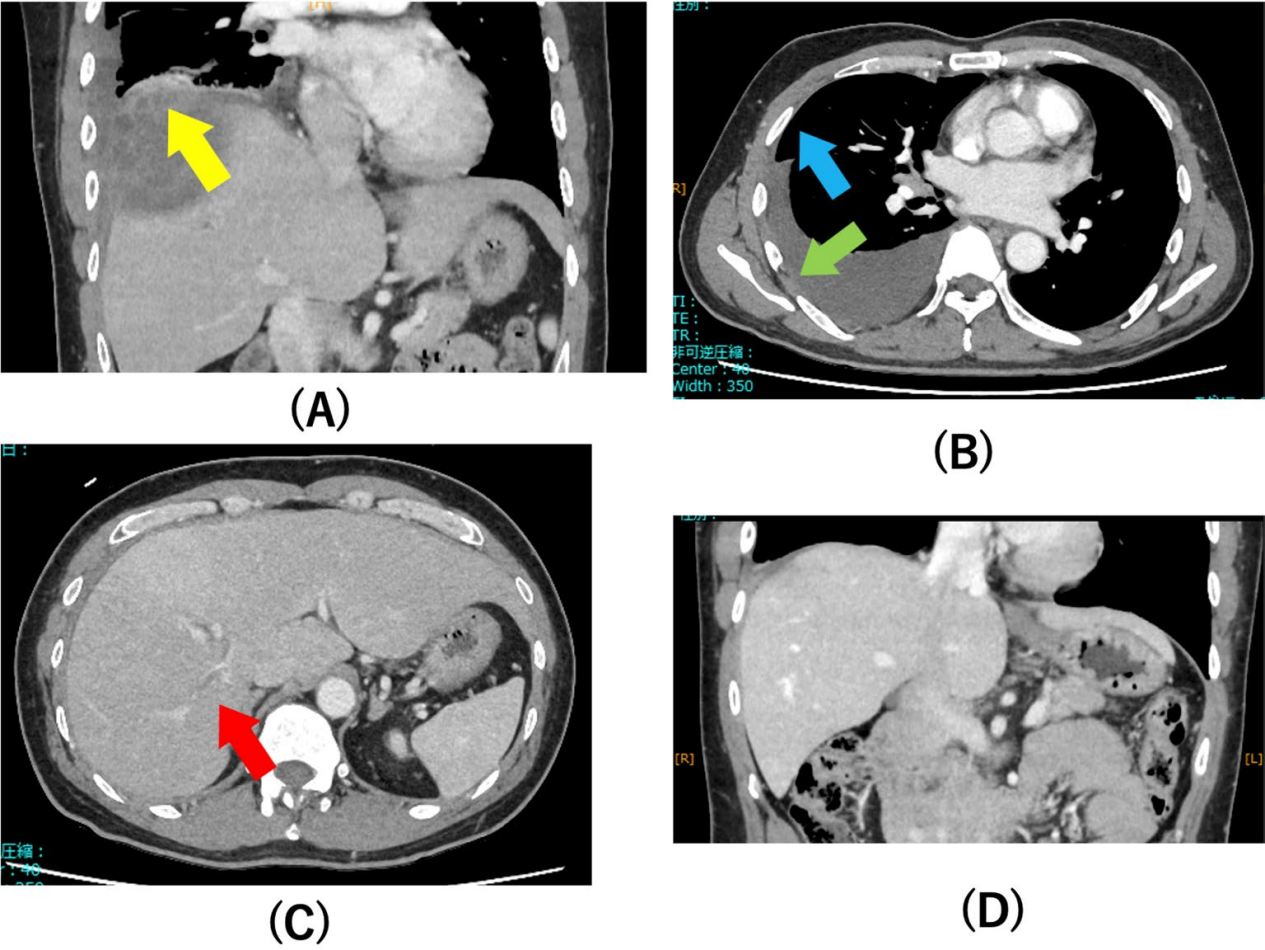
Contrast-enhanced computed tomography (CT) of the thorax and abdomen shows a solitary liver abscess in the right lobe of the liver with loss of diaphragmatic continuity adjacent to the abscess (**a**, yellow arrow), pleural thickening (**b**, blue arrow), enlargement of intercostal muscles (**b**, green arrow) and portal vein branch thrombosis (**c**, red arrow). CT after resolution shows complete restoration of diaphragmatic rupture (**d**)

Contrast-enhanced computed tomography of the chest and abdomen revealed a solitary ring-enhancing lesion in the right lobe of the liver suggestive of a liver abscess. The lesion extended beyond the liver capsule, disrupting the diaphragm (Fig. 1a), with enlargement of the intercostal muscle and pleural thickening on the right side (Fig. 1b). Additionally, a small localized fluid collection was observed in the right pleural cavity. These findings indicated an intrathoracic rupture. However, the pleural fluid volume at this time was insufficient for safe aspiration. In addition, thrombosis of the portal vein branch adjacent to the abscess was observed (Fig. 1c). Microscopic examination of the stool revealed no ova or parasites, and results of antigen testing using *E. histolytica* QuikChek (Techlab, Blacksburg, VA, USA) were negative. However, *E. histolytica* was detected using a multiplex polymerase chain reaction (PCR) FilmArray Gastrointestinal Panel (BioMérieux, France), leading to a definitive diagnosis of amebiasis. Other opportunistic infections were considered less likely based on the normal CD4 count. Although a pyogenic liver abscess was considered, the clinical course and imaging findings were more consistent with those of ALA.

**Fig. 2:**
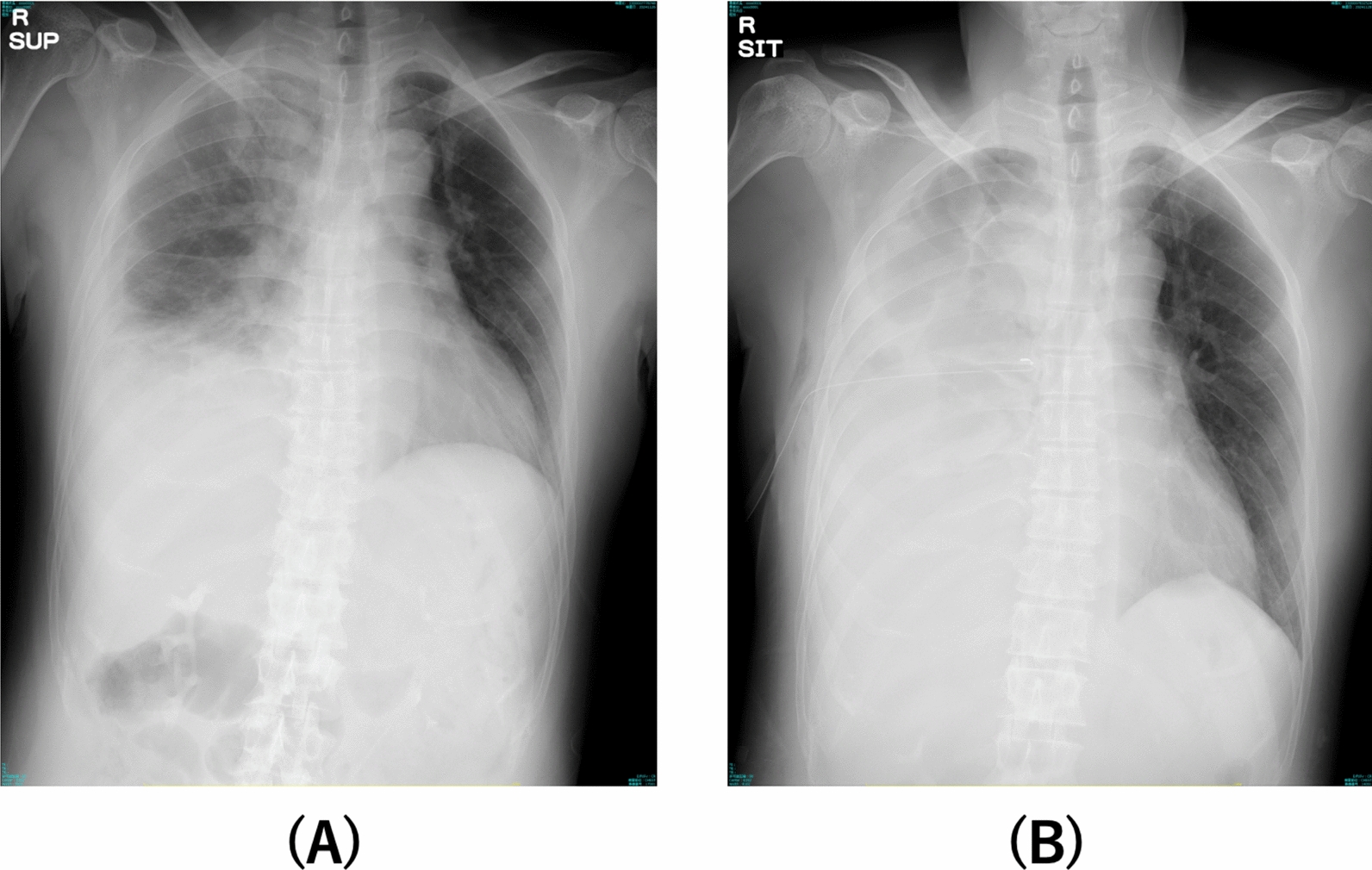
Comparison of chest radiography findings on admission (**a**) and day 3 (**b**) shows significant increase in right pleural effusion

**Fig. 3:**
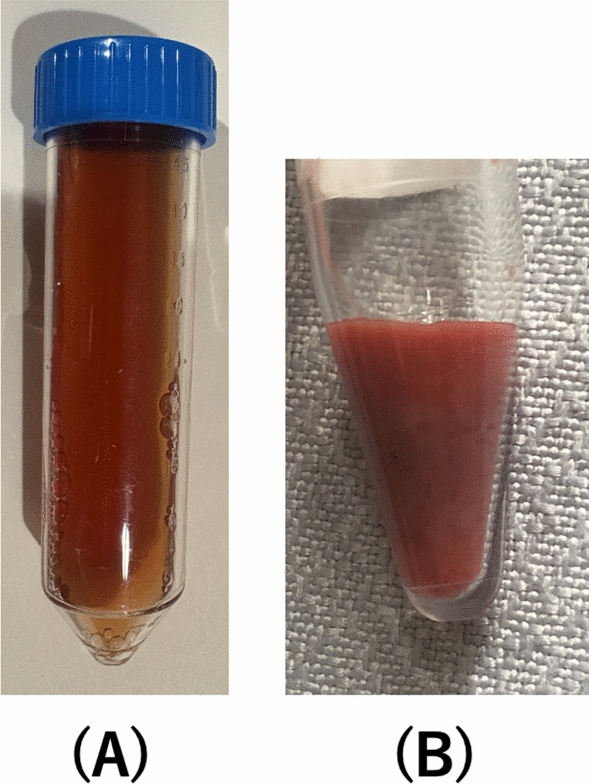
Reddish brown pleural fluid obtained via thoracentesis on day 3 (**a**). Anchovy-sauce-like pus obtained after liver abscess drainage (**b**)

On admission, oral metronidazole (1500 mg per day) was initiated. On day 2, the oxygen demand increased (5 L/min via nasal cannula), and chest radiography revealed worsening of the right pleural effusion (Fig. 2). Thoracentesis yielded a reddish-brown pleural fluid (Fig. 3a). Owing to concerns about secondary bacterial infection or biliary tract involvement, empiric antibacterial therapy (ceftriaxone 2 g/day, intravenously) was initiated, and the metronidazole dose was increased to 2,250 mg per day. On day 3, owing to further clinical deterioration, drainage catheters were placed in the pleural cavity and hepatic abscesses. The liver aspirate had an anchovy sauce-like appearance (Fig. 3b). Microscopic and pathological examinations with hematoxylin–eosin and periodic acid-Schiff staining of the pleural and liver aspirates showed no visible *E. histolytica*.

To confirm the diagnosis, we performed quantitative PCR (qPCR) using TaqMan probes targeting the small subunit rRNA gene (GenBank: X64142) [12], according to a previous report [13]. Thermal cycling included an initial denaturation at 95 °C for 30 s, followed by 40 cycles at 95 °C for 5 s, and 62 °C for 30 s. Cycle-threshold values were analyzed using QuantStudio™ Design & Analysis software (Thermo Fisher Scientific, Waltham, MA, USA). The cycle-threshold values for the stool, liver-abscess aspirate, and pleural fluid were 24.5, 24.3, and 31.7, respectively, which confirmed the presence of *E. histolytica* DNA.

After drainage, the patient’s clinical and laboratory parameters improved. Bacterial cultures of the pleural and liver fluids were negative, and antibacterial therapy was terminated. On day 11, colonoscopy revealed no intestinal lesions, and the qPCR assay of the intestinal fluid was negative. Metronidazole treatment was continued for 14 days.

The hepatic drain was removed on day 17, and the chest drain was removed on day 22 after confirming reduced drainage output and improved imaging findings. On day 22, a follow-up qPCR assay of the pleural fluid revealed a cycle-threshold value of 35.9, suggesting a decrease in pathogen load. The patient was discharged on day 25 of hospitalization.

At the follow-up 5 days later, CT showed liver abscess shrinkage; however, portal vein thrombosis persisted. Therefore, edoxaban (60 mg/day per oral for 1 month) was initiated. In addition, paromomycin (1500 mg/day per oral) was administered for 10 days to eradicate any residual intestinal cysts. At the 3-month follow-up, CT showed complete resolution, with no diaphragmatic defects or portal vein thrombosis (Fig. 1d). The patient was asymptomatic, and D-dimer levels had normalized. No recurrence was observed at the 6-month follow-up.

## Discussion and conclusions

In this patient, ALA with intrapleural rupture was diagnosed based on imaging findings, despite the misleading symptom of right shoulder pain. Imaging findings play a crucial role in the diagnosis of ALA and its complications. Contrast-enhanced CT typically reveals a solitary low-attenuation lesion with peripheral rim enhancement and surrounding edema [14]. In HIV-positive MSM with a preserved CD4 count, the presence of a solitary ring-enhancing liver lesion strongly supports the diagnosis of ALA. Signs of rupture include diaphragmatic disruption, pleural thickening, and increased pleural effusion [15–17]. In our patient, contrast-enhanced CT clearly demonstrated extension of the liver abscess beyond the hepatic parenchyma into the thoracic cavity, with diaphragmatic penetration. Additionally, postprocedural imaging revealed a hepatic drain traversing the pleural space, further supporting the diagnosis of intrathoracic rupture.

Intrapleural rupture is diagnosed based on imaging findings [10] or microscopic examination of the pleural fluid [14, 17, 18]. However, in patients with ALA with pleural effusion, distinguishing between reactive pleural effusion and true intrapleural rupture is essential for prognosis. In a case series that included 501 patients, the mortality rate in patients with reactive pleural effusion and rupture was 2.3% and 11.4%, respectively [10]. In our patient, rapid pleural fluid accumulation and hypoxemia necessitated urgent drainage. The diagnosis of intrathoracic rupture was primarily based on radiological evidence of diaphragmatic disruption and transdiaphragmatic extension of the abscess into the thoracic cavity. Because circulating DNA may appear in the pleural fluid, we interpreted the qPCR findings as supportive evidence. Early distinction allowed for prompt treatment and likely improved clinical outcomes.

Microscopic examination of pleural or liver aspirates has low sensitivity (< 10%) for detecting *E. histolytica* [10, 19], and was negative in this case. Serological tests for anti-amebic antibodies are generally sensitive (70–100%) and specific (> 85%) but may be unreliable in acute colitis, with false negatives in the early phase (within 7–10 days) [2]. Furthermore, in endemic regions, owing to persistent antibody titers, distinguishing current from past infections is difficult. In contrast, PCR offers high sensitivity and specificity, even when microscopy is negative. In our case, PCR rapidly identified *E. histolytica* in the pleural fluid, liver-abscess aspirate, and stool. The cycle-threshold value of the pleural fluid (32.7) was higher than those of the stool (24.5) and liver-abscess aspirate (24.3). PCR testing for *E. histolytica* in pleural effusions from patients with ALA has not been reported, and the association between PCR positivity and rupture is unclear. A previous study detected *E. histolytica* DNA in the blood, urine, and saliva of patients with ALA [20], suggesting that circulating DNA may be present without local invasion. Thus, PCR positivity in the pleural fluid alone is not sufficient to diagnose thoracic ruptures. Further research is needed to confirm the usefulness of PCR for detecting intrapleural ruptures.

The first-line therapy for ALA is metronidazole (500–750 mg thrice daily for 7–10 days). Invasive interventions such as drainage or surgical resection are usually unnecessary but may be warranted in the following high-risk conditions: lack of clinical improvement after 5–7 days of treatment, large abscess (> 5 cm), or involvement of the left hepatic lobe. Surgical treatment is indicated in cases of gastrointestinal bleeding or toxic megacolon [21]. In the past two decades, eight cases of ALA with confirmed intrathoracic rupture have been reported (Table 1) [15–18, 22–25] and diagnosed via imaging and microscopy. All patients underwent thoracostomy or thoracentesis plus metronidazole therapy. Our case emphasizes the importance of careful radiographic assessment and timely drainage when rupture is suspected.

**Table 1:** Cases of ALA with thoracic rupture

Reference	Year	Age	Sex	Country	Diagnosis	Treatment	Duration	Surgical intervention	Outcome
This case	2025	46	Male	Japan	PCR	MNZ 2250 mg/day → paromomycin	14 days	Thoracostomy	Alive
[22]	2023	30	Female	India	Radiographic	MNZ 2500 mg/day + CTRX 1 g q 12 h	NA	Thoracostomy	Dead
[18]	2022	35	Male	USA	Microscopic	MNZ 2250 mg/day	≥ 20 days	Thoracentesis	Alive
[15]	2022	39	Male	Mexico	Radiographic	MNZ 2250 mg/day + CFPM 2 g q 12 h	NA	Thoracostomy + VATS	Alive
[17]	2021	23	Male	Indonesia	Microscopic	MEPM 1 g q 8 h + MNZ 2250 mg/day	≥ 14 days	Thoracostomy	Alive
[23]	2020	26	Male	Mexico	Radiographic	MNZ + CTRX	NA	Thoracostomy	Alive
[24]	2020	51	Male	Sri Lanka	Radiographic	MNZ + CTRX	6 weeks	Thoracostomy	Alive
[16]	2012	27	Male	China	Radiographic	MNZ 1500 mg/day → iodoquinol	21 days	Thoracostomy	Alive
[25]	2009	57	Male	France	Microscopic	MNZ 1500 mg/day	10 days	Thoracentesis	Alive

*ALA* amebic liver abscess, *MNZ* metronidazole, *CTRX* ceftriaxone, *VATS* video-assisted thoracic surgery, *CFPM* cefepime, *MEPM* meropenem, *USA* United States of America

Portal vein thrombosis is observed in up to 63% of patients with ALA [26], and thrombosis of the hepatic vein and inferior vena cava has been reported (Table 2) [27–37]. Management strategies vary; some patients require thrombolysis owing to hemodynamic compromise, whereas others resolve without anticoagulation. In our patient, edoxaban was initiated after stabilization, considering the risk of bleeding during pleural drainage. Anticoagulation should be tailored to balance the thrombotic risk and bleeding potential.

**Table 2:** Cases of venous thrombosis with ALA

Reference	Year	Age	Sex	Country	Site	Treatment	Outcome
This case	2025	46	Male	Japan	HV	Anticoagulation	Alive
[27]	2024	61	Male	USA	PA	Anticoagulation	Alive
[28]	2020	44	Male	Italy	PV	None	Alive
[29]	2017	43	Male	Canada	IVC, HV, CIV	Thrombolysis, Anticoagulation	Alive
[30]	2015	43	Male	USA	HV, IVC, PA	Anticoagulation	Alive
[31]	2015	20	Female	UK	PV	None	Alive
[32]	2014	3	Female	India	RA, IVC	Thrombectomy, Anticoagulation	Alive
[33]	2013	2	Male	India	RA, IVC	Thrombectomy, Anticoagulation	Alive
[34]	2013	6	Male	India	RA, IVC, HV	Thrombectomy	Alive
[35]	2012	47	Male	India	IVC	None	Alive
[36]	2011	24	Male	India	IVC	None	Alive
[36]	2011	21	Male	India	IVC	None	Alive
[36]	2011	61	Male	India	IVC	None	Alive
[37]	2008	57	Male	India	HV, IVC	None	Alive

*ALA* amebic liver abscess, *IVC* inferior vena cava, *HV* hepatic vein, *PA* pulmonary artery, *CIV* common iliac vein, *PA* pulmonary artery, *RA* right atrium, *USA* United States of America

In this patient, radiological imaging revealed diaphragmatic disruption and abscess extension, providing decisive evidence. The rapid progression of pleural effusion and need for thoracic drainage emphasize the importance of early diagnosis and treatment. Clinicians should monitor thoracic signs in ALA, particularly if respiratory symptoms worsen, and act quickly. As a single case report, these observations may not be generalizable, but they highlight important diagnostic and therapeutic considerations.

## Data Availability

Data sharing is not applicable to this article as no datasets were generated or analyzed during the study.

## Abbreviations

ALA: Amebic liver abscess
CT: Computed tomography
MSM: Men who have sex with men
PCR: Polymerase chain reaction
qPCR: Quantitative polymerase chain reaction
